# Long-term outcome in relationship to neonatal transfusion volume in extremely premature infants: a comparative cohort study

**DOI:** 10.1186/1471-2431-11-48

**Published:** 2011-05-28

**Authors:** Jeannette S von Lindern, Chantal M Khodabux, Karien EA Hack, Ingrid C van Haastert, Corine Koopman-Esseboom, Paul HT van Zwieten, Anneke Brand, Frans J Walther

**Affiliations:** 1 Division of Neonatology J6-S, Department of Pediatrics, Leiden University Medical Center, PO BOX 9600, 2300RC Leiden, the Netherlands; 2 Department of Pediatrics, Groene Hart Ziekenhuis, Gouda, the Netherlands; 3 Sanquin Blood Bank South-West Region, Leiden, the Netherlands; 4 Department of Obstetrics, University Medical Center Utrecht, Utrecht, the Netherlands; 5 Department of Neonatology, Wilhelmina Children's Hospital, University Medical Center Utrecht, Utrecht, the Netherlands; 6 Department of Immuno-haematology and Transfusion, Leiden University Medical Center, Leiden, the Netherlands; 7 Juliana Children's Hospital, the Hague, the Netherlands

## Abstract

**Background:**

In premature born infants red blood cell (RBC) transfusions have been associated with both beneficial and detrimental sequels. Upon RBC transfusion, improvement in cerebral blood flow and oxygenation have been observed, while a more liberal transfusion policy may be associated with a better developmental outcome. The effect of the transfusion volume on long-term outcome is not known.

**Methods:**

Observational follow-up study of a cohort of extremely premature born infants, treated in 2 neonatal intensive care units using a different transfusion volume (15 ml/kg in Unit A and 20 ml/kg in Unit B). The primary outcome was a composite of post discharge mortality, neuromotor developmental delay, blindness or deafness, evaluated at a mean corrected age (CA) of 24 months related to the transfusion volume/kg bodyweight administered during the postnatal hospital stay.

**Results:**

Despite the difference in transfusion volume in clinically comparable groups of infants, they received a similar number of transfusions (5.5 ± 3.2 versus 5.5 ± 2.3 respectively in Unit A and B). The total transfused volume in unit A was 79 ± 47 ml/kg and 108 ± 47 ml/kg in unit B (p = 0.02). Total transfused RBC volume per kg bodyweight was not an independent predictor of the composite outcome (p = 0.96, OR 1.0 (CI 0.9-1.1).

**Conclusion:**

There was no relationship between the composite outcome at 24 months CA and transfusion volume received during the post natal hospital stay. As there was no clinical advantage of the higher transfusion volume, a more restrictive volume will reduce total transfusion volume and donor exposure. Future research on the optimal transfusion volume per event to extreme preterm infants should include larger, prospective studies with a longer follow-up period through to childhood or even adolescence.

## Background

There is ongoing uncertainty whether transfusion of red blood cells (RBC) in the neonatal period influences the clinical outcome and development of premature infants. In the international literature there is discussion about the optimal transfusion volume and trigger [1].

We previously published a study comparing two transfusion dosages of the same RBC product in two Dutch tertiary care neonatal units (NICUs) using the same transfusion trigger protocols [2]. No difference in short-term outcome and mortality was observed in extremely and very preterm infants when a dose of 15 ml/kg had been administered compared to a volume of 20 ml/kg bodyweight.

Little is known about the long-term follow-up of extremely premature infants after RBC transfusion. Only a few randomized trials have been published on long-term outcome in premature infants with difference in transfusion practice [3,4]. However, these were studies (the PINT trial and the Iowa trial) comparing liberal and restrictive transfusion triggers, exposing the infants in the restrictive group to the possible risks of a low hemoglobin level [5,6]. In sequel to our study on short-term outcome we also wanted to compare long-term outcome in the same group of extremely premature infants treated with different transfusion volumes but with otherwise comparable transfusion triggers and transfusion products.

In our follow-up study we evaluated post discharge mortality, neuromotor developmental outcome and disabilities in a cohort of extremely premature born infants born before 28 gestational weeks in two Dutch tertiary NICUs.

## Methods

### Ethics

In the Netherlands no ethical approval is required for this type of research as no new intervention or treatment is studied. All collected data were anonymous.

### Study population

This is a follow-up study of a cohort of extremely premature infants born before 28 gestational weeks that participated in a previous study on transfusion practice and short-term outcome in two Dutch NICUs. All infants were transfused using the same transfusion triggers and with a similar RBC blood product with only a different volume per event, i.e. 15 and 20 ml/kg bodyweight. The study design was previously described [2]. The primary outcome measure was a composite of post discharge mortality, neuromotor developmental outcome and disabilities.

### Transfusion guideline and product

All infants were transfused according to the Dutch consensus for blood transfusion 2004 [7]. The recommended transfusion triggers vary with postnatal age, degree of illness and need for respiratory support:

- Hb <8 mmol/l (13 g/dl) (hematocrit (Hct) range 0.38-0.40 l/l) capillary (or <7 mmol/l arterial (11 g/dl) (Hct 0.32-0.35 l/l)): the first 24 hours after birth in all infants with clinical symptoms of anemia (tachycardia, supplemental oxygen need, apnoea, bradycardia); in all infants on mechanical ventilation or severely ill.

- Hb <7 mmol/l (12 g/dl) (Hct 0.32-0.35 l/l) capillary: reasonably stable infants with cardio-respiratory problems (patent ductus arteriosus, apnoea, bronchopulmonary dysplasia, need for supplemental oxygen).

- Hb <6 mmol/l (10 g/dl) (Hct 0.27-0.30 l/l) capillary: stable premature infants with a postnatal age <4 weeks.

- Hb <4.5 mmol/l (7 g/dl) (Hct 0.2-0.23 l/l) capillary: stable infants with a postnatal age >4 weeks if there are no signs of anemia (apnoea, tachycardia, poor weight gain, poor feeding). In case of symptomatic anemia, transfusion is recommended at a higher threshold.

Transfusion volume per kg bodyweight was different between the two hospitals; 15 ml/kg in Unit A and 20 ml/kg in Unit B. This transfusion volume was part of the standard practice in the hospitals and was not chosen for study purposes. The same transfusion product was used in both hospitals. All products consisted of pre-storage filtered RBC stored in additive solution Saline Adenine Glucose Mannitol (SAG-M) (maximum storage time 35 days), with a hematocrit of 0.58 ± 0.05 l/l. The products were irradiated with 25 Gy less than 24 hours before transfusion.

Preventative measures for anemia of prematurity were not standardised. Erythropoietin was not used in the study units. Iron supplementation was started 6 to 8 weeks after birth if there had been no previous RBC transfusion. After RBC transfusion iron supplementation was postponed for four weeks because of the assumed iron load given with the transfusion.

### Data collection and outcome parameters

Infants with a syndrome or congenital/hereditary anomaly known to cause a neuromotor developmental delay were excluded from analysis. Data on neuromotor developmental outcome, major disabilities (deafness, blindness) and survival at a mean (± SD) corrected age (CA) of 24 ± 3.4 months were obtained from each child's outpatient follow-up physician, child-psychologist and/or pediatric physiotherapist, who were all trained in neonatal follow-up. Developmental examination was done in different hospitals. The children were assessed with various instruments depending on the hospital of follow-up. Instruments used were the Dutch 2^nd ^version of the Bayley Scales of Infant Development-II (BSID-II-NL) [8], the Griffiths Mental Development Scales [9], Alberta Infant Motor Scale (AIMS) [10], and the Hempel [11], Touwen [12], and van Wiechen [13] assessments of neuromotor development. The children were classified as normal, mildly delayed or severely delayed, using the cut-off values of each test, classifying severe neuromotor developmental delay as a score of more than 2 SD below average and mild neuromotor developmental delay as a score 1 to 2 SD below the mean. Non-cooperative children received a general assessment by the physician, pediatric physiotherapist and/or child-psychologist. A parental questionnaire was sent to the parents of the children if follow-up at age 2 years was unknown by any of the previously mentioned professionals.

Our primary outcome was the composite of post discharge mortality, severe hearing or visual impairment, or neuromotor developmental delay at 24 months CA. Visual impairment was defined as <20/200 of best eye, hearing impairment was defined as the need for a hearing aid or cochlear implant. Neuromotor developmental delay was defined as a score more than 1 SD below the mean. The definition for bronchopulmonary dysplasia (National Institutes of health Consensus, USA) was used as described by Ehrenkranz et al [14]. For retinopathy of prematurity the revised international classification was used [15]. Intraventricular hemorrhage was graded according to Volpe [16].

### Statistical Analysis

All variables were analyzed by univariate analysis for continuous variables and Chi-Square or Fishers exact probability test for nominal variables. Backward step wise logistic regression analysis was used for the independent effect of the following factors: hospital (representing transfusion dose and unknown factors), number of RBC transfusions, total transfused RBC volume per kg bodyweight, gender, gestational age, birth weight, CRIB II score and Apgar- score at 5 minutes. (SPSS 17 Chicago, United States of America). A p-value of less than 0.05 was considered significant.

## Results

Eighty-seven extremely premature infants were included in our earlier cohort study (44 in Unit A, 43 in Unit B). Four infants died the first day of life and were excluded from this follow-up study (1 lung hypoplasia, 1 massive pulmonary hemorrhage, 1 severe perinatal asphyxia and 1 cause unknown). None of these children had received a RBC transfusion. Twelve other patients died in the neonatal period for various reasons; they had all received RBC transfusions. They were also excluded from follow-up. Seventy-one of the 87 infants (82%) left the hospital alive (in total 9 in unit A and 7 in unit B died (p = 0.78). There were no deaths after discharge from the units. One other patient was excluded from follow-up analysis because of a severe myopathy. Three children were lost to follow-up in the first year of life; one due to emigration, of the other two (twins) the reason is unknown, leaving 67/70 (96%) of the eligible infants surviving the post-natal period for neuromotor developmental follow-up.

There were no statistically significant differences when comparing the baseline characteristics of the patients from both units (Table 1). The number of transfusions administered was similar, 5.5 ± 3.2 (Unit A) versus 5.5 ± 2.3 (Unit B) respectively. As the volume per transfusion event per center differed, the mean total volume transfused in unit A and B was significantly different (79 ± 47 ml/kg versus 108 ± 47 ml/kg (p = 0.02) respectively). Neuromotor development was evaluated at a mean 24 ± 3.4 months CA. One infant had a severe visual impairment and one child had severe hearing loss. Forty-seven out of 67 (70.1%) infants showed normal neuromotor development for corrected age. Seventeen infants (25.4%) had a mild developmental delay and three infants were severely impaired (4.5%).

**Table 1:** Characteristics of patients per unit included in follow-up study

	Total (n = 67)	Unit A (n = 31)	Unit B (n = 36)	p-value
Male, n	61% (41)	74% (23)	50% (18)	0.05

Gestational age, weeks mean SD	26 6/7 ± 4/7	26 6/7 ± 4/7	26 6/7 ± 4/7	0.65

Birth weight, grams mean SD	900 ± 187	906 ± 178	895 ± 197	0.59

AS 5 minutes, median (IQR)	8 (7-9)	8 (7-9)	8 (7-9)	0.88

IVH ≥ grade 3	9% (6)	13% (4)	6% (2)	0.40

BPD≥ moderate	35% (23)	29% (9)	40% (14)	0.35

ROP ≥ grade 2	12% (8)	13%(4)	11% (4)	1.0

Total number of transfusions, mean SD	5.5 ± 2.7	5.5 ± 3.2	5.5 ± 2.3	0.98

ML/per kg RBC administered, mean SD	95 ± 49	79 ± 47	108 ± 47	0.02

Composite outcome	31% (21)	29% (9)	33% (12)	0.71

AS = Apgar score, IVH = intraventricular hemorrhage, BPD = bronchopulmonary dysplasia, ROP = retinopathy of prematurity, RBC = red blood cells, composite outcome = post discharge mortality, impaired neuromotor development, deafness or blindness

There was no statistically significant difference in the transfused volume for the primary outcome (composite of post discharge mortality, neuromotor developmental delay, blindness or deafness) compared to children with a normal outcome (105 ± 52 ml versus 90 ± 47 ml respectively) (p = 0.96, OR 1.0 (0.9-1.1). In multivariate analysis none of the tested variables reached statistical significance for an independent association with the composite outcome (Table 2).

**Table 2:** Predictors of the composite outcome of neuromotor developmental delay, post-discharge mortality, blindness or deafness

	P-value	OR	CI of OR (95%)
Hospital	0.8	1.6	0.1-24.7

Number of RBC transfusions	1.0	1.0	0.2-4.8

ML/kg RBC transfused	1.0	1.0	0.9-1.1

Gender	0.2	0.4	0.0-1.6

Gestational age	0.8	1.0	0.9-1.1

Birth weight	0.4	1.0	1.0-1.0

CRIB II score	0.6	1.0	1.0-1.0

Apgar score 5 min	0.3	1.2	0.8-1.9

RBC = red blood cells

## Discussion

Transfusion of RBC has been associated with negative and positive effects on clinical outcome. Our study showed no significant differences between a smaller and a larger transfusion volume regarding a composite outcome of post discharge mortality, neuromotor developmental outcome and disabilities at 2 years CA. This would imply that a smaller transfusion volume rather has advantages (generally limiting donor exposure and costs), than negative effects on neuromotor developmental outcome. It is possible that no effects were seen because the difference in transfusion volume per transfusion event was not large enough. On the other hand, the total transfusion volume/kg during the post natal hospital stay was significantly different in the two NICUs. Several studies associated RBC transfusion with the development of retinopathy of prematurity [17-20] and chronic lung disease [21-23]. It is hypothesized that iron overload, caused by multiple RBC transfusions, increases oxidative stress leading to free radical-induced injury to the premature retina and developing lungs [24,25]. However, anemia has been associated with negative effects as well and in particular the brain may be susceptible to low hemoglobin levels. Bell et al, in a randomized study, found a statistically significant higher incidence in the number of infants with intracranial hemorrhage grade IV or periventricular leukomalacia when a restrictive transfusion threshold was applied as compared to a more liberal threshold [5]. On the other hand, neither Chen [24] nor Kirpalani (PINT trial) [6] found a statistically significant difference in the occurrence of (severe) intracranial pathology. However, the follow-up study by Whyte et al. of infants included in the PINT trial, suggested that preterm infants treated with a more liberal transfusion regime may have a better developmental outcome [4]. The premature infants included in this multicenter PINT trial were analyzed at 18-21 months CA with regard to mortality, cerebral palsy, severe visual impairment, hearing-loss, a Bayley- Mental Developmental Index (MDI) score <70, and a composite of these variables. Although there was no statistically significant difference in composite outcome (45% in the restrictive and 38% in the liberal group), the difference in cognitive delay (MDI score <70) approached statistical significance in favor of the liberal group. A post-hoc analysis with cognitive delay redefined (MDI score more than 1SD below the age-standardized mean) showed a significant difference favoring the liberal threshold group. This suggests that premature infants may benefit from a higher hemoglobin threshold for transfusion [4]. In the study by Nopoulos et al. on the brain structure of 12 year old children previously enrolled in the Iowa trial by Bell and colleagues, only 44 of the 100 children participated [3]. The children who were exposed to the liberal transfusion threshold appeared to have a substantially smaller intracranial volume compared to the children transfused according to the restrictive guideline. Nopoulos mentions unpublished data on long-term cognitive follow-up showing (non-significant but) overall poorer outcome in the group of liberal transfused children.

Another recent study has shown that RBC transfusions increase cerebral oxygenation, thereby decreasing the risk of tissue hypoxia [26]. Mercer et al. showed that premature male infants after delayed cord clamping, had a better developmental outcome at seven months CA compared to infants after immediate cord clamping [27]. It is conceivable that upon transfusion of a larger volume, an increase in cerebral flow and better oxygenation can be obtained with a similar effect as described by Mercer.

In transfusion guidelines, the range of recommended RBC volume per kilogram bodyweight is rather wide, 10-20 ml/kg. Given the variation in hematocrit between used RBC products, the guidelines result in a huge variation in actually transfused RBC.

We observed no influence of transfused volume RBC per kg bodyweight on a composite of impaired neuromotor development, post discharge mortality and deafness or blindness in extremely preterm born infants evaluated at 24 months CA. This finding remained unchanged after logistic regression analysis correcting for other factors relevant for outcome.

The two Dutch NICUs participating in this study used the same transfusion guideline, transfusion trigger and transfusion product, which virtually excludes important differences in degree of anemia between infants. Our study has its limits being a retrospective, non-randomized trial. The neuromotor development was assessed using various different tests. In Unit B all children were assessed by the same special educator who is also a pediatric physiotherapist (ICvH) using the same test (BSID-II-NL) in 95% of the children. The children from Unit A were assessed using various validated tests, performed in different hospitals by different professionals. It may be an incentive to perform a larger, prospective randomized controlled trial, focusing not only on the transfusion trigger but also on the total transfused RBC volume to recommend an optimal transfusion trigger and dose.

## Conclusion

A high total transfused volume of RBC per kg was not correlated with the composite outcome of impaired neuromotor developmental outcome, post discharge mortality, blindness or deafness when analyzed at 24 ± 3.4 months CA, hereby questioning the previously presumed possible detrimental effects of RBC transfusions.

### Recommendations

Due to differences in neonatal transfusion practice, conclusions on the effect of maintaining a higher Hb value by the use of liberal transfusion triggers or a larger volume of RBC per transfusion are not based on strong evidence. A larger transfusion volume may not be associated with deleterious effects, whereas a smaller transfusion volume may limit donor exposure. Through randomized clinical studies, the effect of more liberal or restrictive triggers on long-term outcome can be studied, as well as the combination with a low (10 ml/kg) or high (20 ml/kg) transfusion volume. A longitudinal study to school age or even adolescence looking for instance at academic competencies might give further insight in the effects of different transfusion volumes.

## Competing interests

The authors declare that they have no competing interests.

## Authors' contributions

JSvL and CK made substantial contributions to the study design, data retrieval and analysis and interpretation of the data. Both were involved in writing and revising the manuscript.

KEAH, IcvH, CKE and PHTvZ made substantial contributions to the acquisition of data and critical revision of the manuscript. AB and FJW made substantial contributions to the study design and analysis and interpretation of the data and were involved in revising the manuscript for important intellectual content. All authors have given approval of the final document.

## Pre-publication history

The pre-publication history for this paper can be accessed here:

http://www.biomedcentral.com/1471-2431/11/48/prepub
